# ‘Pericardial hyperechoic ring’ on point-of-care ultrasound during cardiopulmonary resuscitation

**DOI:** 10.1093/ehjcr/ytag776

**Published:** 2026-10-21

**Authors:** Koya Akutagawa, Kensuke Ito

**Affiliations:** Emergency and Trauma Center, Kameda Medical Center, 929 Higashi-cho, Kamogawa, Chiba 296-8602, Japan; Emergency and Trauma Center, Kameda Medical Center, 929 Higashi-cho, Kamogawa, Chiba 296-8602, Japan

**Keywords:** Case report, Acute aortic dissection, Cardiac tamponade, Point-of-care ultrasound, Postmortem computed tomography, Hemopericardium

## Summary

A hyperechoic band along the epicardial surface was identified on point-of-care ultrasound during cardiopulmonary resuscitation. This appearance closely resembled the ‘pericardial hyperdense ring’ described on post-mortem computed tomography and may represent a real-time sonographic analogue of this finding.

## Case description

A 71-year-old man with hypertension and diabetes mellitus fell from a ladder. On arrival of emergency medical services, he was apnoeic and in asystole, and cardiopulmonary resuscitation (CPR) was initiated. Return of spontaneous circulation was not achieved, and he was transported to the emergency department with ongoing CPR approximately one hour after the event.

Point-of-care ultrasound performed during resuscitation from an apical window demonstrated pericardial fluid with a hyperechoic band along the epicardial surface (*Figure 1A*, arrow), clearly demarcated from the surrounding anechoic fluid. The band appeared thicker than expected for epicardial fat and lacked the granular echotexture typical of adipose tissue.

**Figure 1:**
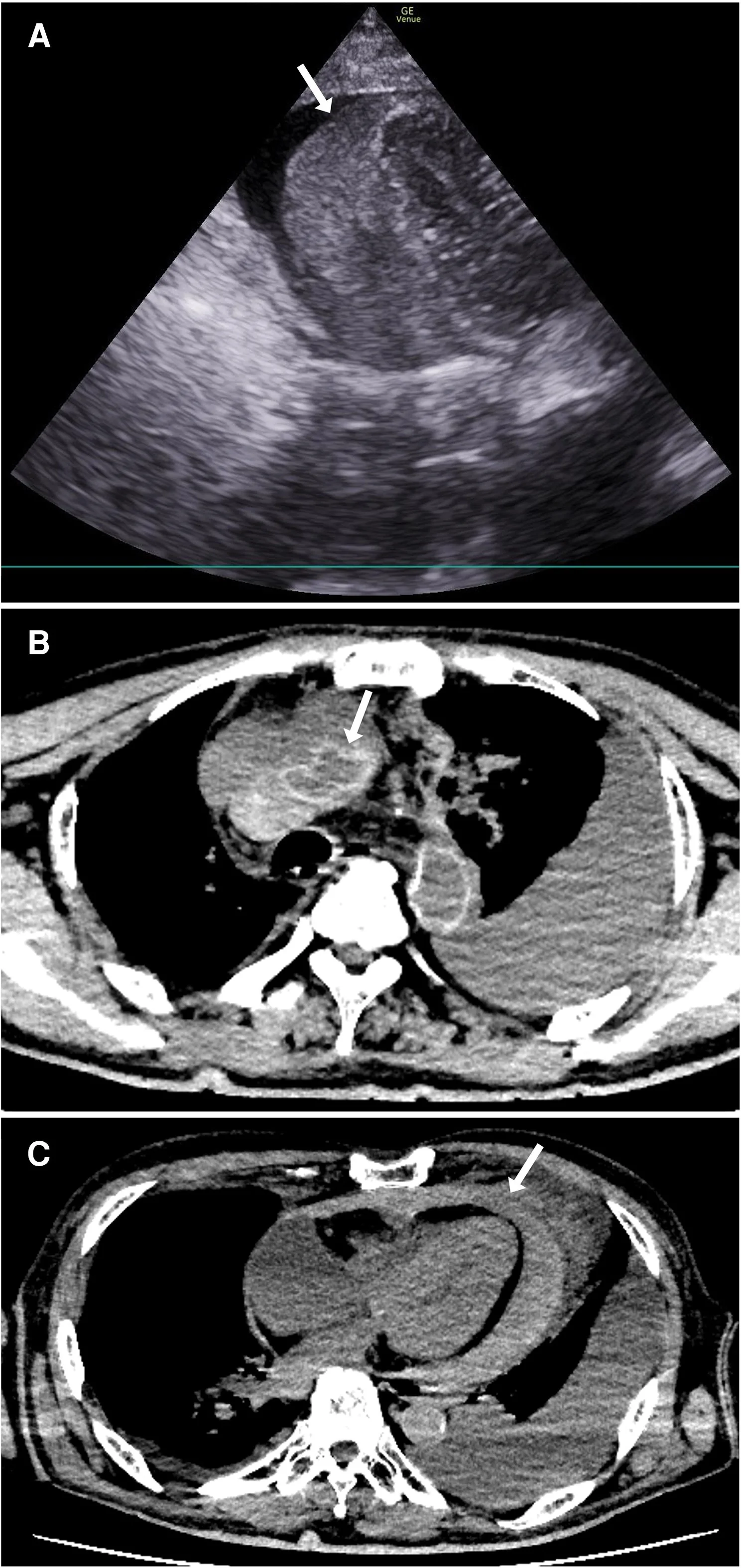
Corresponding point-of-care ultrasound and post-mortem CT findings of haemopericardium in a patient with Stanford type A aortic dissection. (*A*) Point-of-care ultrasound obtained during ongoing cardiopulmonary resuscitation from an apical window shows pericardial fluid with a hyperechoic band along the epicardial surface (arrow), clearly demarcated from the surrounding anechoic pericardial effusion. (*B*) Unenhanced post-mortem computed tomography demonstrates Stanford type A aortic dissection with an intimal flap in the ascending aorta (arrow). (*C*) Unenhanced post-mortem computed tomography at a corresponding axial level demonstrates concentric layering of pericardial blood, consisting of an inner hyperdense layer adjacent to the epicardium (arrow) and an outer relatively hypodense component.

Given the prolonged low-flow state and initial non-shockable rhythm, further resuscitative efforts were considered futile and CPR was terminated. Post-mortem computed tomography (PMCT) revealed a Stanford type A aortic dissection with haemopericardium. An intimal flap was visible in the ascending aorta (*Figure 1B*, arrow). Pericardial blood demonstrated concentric layering with an inner hyperdense component adjacent to the epicardium and an outer relatively hypodense layer (*Figure 1C*, arrow). No traumatic injuries were identified on examination or CT. The patient had no history of cardiac procedures or cardiac devices. Although myocardial infarction cannot be completely excluded, the abrupt clinical course and the presence of type A aortic dissection with haemopericardium on PMCT made myocardial rupture unlikely. Death was attributed to cardiac tamponade secondary to aortic dissection.

On PMCT, a circumferential hyperdense band—termed the ‘pericardial hyperdense ring’—has been described as a marker of sustained antemortem pericardial haemorrhage, including layered haemopericardium such as the hyperdense armoured heart, in contrast to post-resuscitation haemorrhage, which typically demonstrates a fluid–fluid level pattern.^1–3^ The present case suggests that this imaging feature may have a sonographic counterpart, appearing as a ‘pericardial hyperechoic ring’ on point-of-care ultrasound during ongoing CPR. Recognition of this pattern may indicate that haemopericardium preceded cardiac arrest rather than representing a consequence of resuscitative efforts.

## Data Availability

The data underlying this article are available within the article. No additional data are available.
